# Adolescents Perceptions of Pro- and Antitobacco Imagery and Marketing: Qualitative Study of Students from Suva, Fiji

**DOI:** 10.1155/2015/602635

**Published:** 2015-08-25

**Authors:** Gade Waqa, Judith McCool, Wendy Snowdon, Becky Freeman

**Affiliations:** ^1^C-POND, Fiji National University, Fiji; ^2^Social and Community Health, Faculty of Medical and Health Science, University of Auckland, New Zealand; ^3^School of Public Health, Faculty of Medicine, University of Sydney, Australia

## Abstract

*Background*. Many studies examining smoking uptake among young people in the Pacific have not included their exposure to tobacco control promotions in the media in their assessment. This study examines how Fijian students view tobacco and tobacco-related media depictions to gain insight into both drivers of smoking uptake and potential directions for prevention interventions. *Methods*. A sample of thirty Fijian students (15 male and 15 female) aged 14–17 years, was recruited from a Suva school between September and October 2013 and participated in a one-to-one in-depth interview about their views on tobacco use, media consumption patterns and preferences and awareness of tobacco use in media. *Results*. Despite radical developments in access to media, television remains the most popular. Yet, the majority of participants were unaware of any protobacco imagery on television or other entertainment media. Tobacco-related imagery was more likely to be seen in connection with point of sale advertising and branding. The advertising potential of the shop counter was acutely apparent to some participants and this space was considered highly influential. *Conclusions*. Despite the fact that the recently introduced graphic health warnings were generally well received, more can be done to extend the use of media for tobacco control benefits in Fiji.

## 1. Introduction

Tobacco use seriously undermines progress in reducing current and projected rates of noncommunicable disease (NCD) in Pacific Islands region [1, 2]. According to the WHO, approximately 55% of male and 17% of female adults in Fiji are smokers [3]. Consistent with international evidence on the relationship between adult and youth tobacco use [4], tobacco use is high among young Fijians [3]. Up to 71% of young people had tried a cigarette before the age of 14 years. Fifty-six percent reported being exposed to second-hand smoke regularly and 12% used tobacco frequently (one or more days in past week). With the support of the WHO Framework Convention on Tobacco Control (WHO FCTC) [5], the WHO NCD Roadmap [6], and the Tobacco Free Pacific 2025 [7], the Pacific has mandated reducing tobacco use as a priority for reducing the regional burden of NCD [7]. Translating political endorsement into policy that advances social change is a continuing challenge for all countries. The Pacific Islands countries are facing the compounded issue of local production and distribution of tobacco products, a young and growing population, and, in some contexts, delayed tobacco control response.

Much like the rest of the Pacific Islands region, Fiji faces the ubiquitous challenge of implementing cost effective tobacco use prevention and cessation policies that will result in significant declines in smoking prevalence [7, 8]. Tobacco control “best buys” are promoted as ideal, broad-reach measures that are most likely to reduce tobacco consumption and prevalence [9]. These measures include establishing and enforcing smokefree environments, warning about the risks of tobacco use, increasing the cost of tobacco (taxation), offering quit smoking assistance, and enforcing bans on tobacco advertising and promotion [10]. Fiji has successfully implemented many of these best practice approaches. Since October 2013, all tobacco products, including duty free, include a graphic and text warning which cover 30% of the front and 90% of the back of packs. Images were developed from local sources. Accompanying text is presented in English on the front and bilingual (iTaukei and Hindi) on the back of packs. Smokefree environments are being extended to include hospitals, government offices, and quarters and some villages, and local mass media education campaigns have been developed in conjunction with these changes. The primary mechanism for delivery of the media is posters and billboards at point of sale.

Mass media based campaigns have formed an important facet of any comprehensive tobacco campaign [11, 12]. Investment in antitobacco mass media campaigns has proven to be successful in reducing smoking prevalence in high income countries [13] where they promote quitting and reduce uptake and decrease the social acceptability of smoking. However, these claims are based on evidence of impact in higher income countries where there is also likely to be a broader investment in tobacco control, greater exposure to the campaigns, and some capacity available to evaluate the impact [11]. Evidence from low and middle income or emerging economies is far less available, despite good indication that countries in these regions are central to tobacco industry marketing [14].

Exposure to mass media antitobacco campaigns alongside protobacco imagery is a likely scenario for LMIC and emerging economies [15]. Previous work identified that although 14 of the 22 PICTs have signed and ratified the WHO FCTC, progress has been hampered by the challenges of implementation and enforcement of the key action areas [16]. Tobacco industry interference remains rife in the region [17] and British American Tobacco has a live presence in Fiji. The advent of new media and tobacco industry exploitation of novel promotional opportunities is a current challenge to global tobacco control efforts [18].

We argue that the social worlds that, consistent with international trends, young people in Fiji occupy are or will become intrinsically influenced by their access to globalised digital media [15, 19]. Low and middle income countries are increasingly gaining access to mobile and Internet connectivity [20] and the implications for this on tobacco control and promotions is important to monitor [19, 21]. However, what drives tobacco use in any population is never a single entity; perceptions of influences on youth tobacco use variously attribute cause to social/environmental and individual determinants or, more accurately, a synergy of several domains [22, 23]. In the present study, we question both the role of family and the environment, particular the media environment, as drivers to tobacco use among young Fijians. We specifically focus our attention on young people in context as being a sensitive barometer of social change. If tobacco control efforts are to be effective they need to be relevant to and resonate with young people [24]. Similarly, they need to be based on recent evidence from their own local communities and not solely extrapolated from international efforts or larger scale surveys [25, 26].

Previous work indicates that young people are a sensitive barometer of social mood and change [27]. Tapping into how young people perceive tobacco in the context of their everyday lives provides insight into current and anticipates challenges for tobacco control in Fiji. Current evidence on the drivers to smoking uptake and the relevance of media and other drivers will assist to strengthen Fiji response to the WHO FCTC Article 13 and contribute actively to supporting the target of a Tobacco Free Pacific (by) 2025. This paper examines how Fijian students view tobacco and tobacco-related media depictions to gain insight into both drivers of smoking uptake and potential directions for prevention interventions.

## 2. Methods

Ethical approval for this study was obtained from the College Health Research Ethics Committee, the Fiji National Research Ethics Review Committee, the Fiji Ministry of Education, Deakin University Health Research Ethics Committee, and the University of Auckland Human Participants Ethics Committee. In-depth interviews were conducted with 30 individuals aged between 14 and 17 years between September and October 2013. Students were recruited from a single class in a mixed gender, ethnicity, and socioeconomic indicator high school in central Suva. The school was selected by the Ministry of Education on the basis of central location and mixed ethnic composition. The interviewer visited the school to discuss the research purpose and method of data collection with the school principal and teachers. It was confirmed that up to 30 students (15 males and 15 females) who provided informed consent from their parents would be invited to participate from a single class. Students who were able to provide informed consent from their parents were then interviewed. Interviews were conducted by a trained interviewer in English. Interviews lasted between 30 and 50 minutes and were conducted during school hours. Consent to audio-record the interviews was sought from each participant. During the start of the interview, students were reminded of protocol for the interview and assured of the confidentiality and anonymity and voluntary nature of the interview and the data analysis. Students were invited to participate in a one-to-one in-depth interview about their views on tobacco use, media consumption patterns, and preferences and awareness of pro- and anti-tobacco media.

Interviews were transcribed verbatim before being provided to the research team for analysis. Firstly the interviews were read and reviewed to generate a first level of coding for the preliminary analysis. A coding framework was developed that reflected the dominant or most pervasive themes or issues that were raised by the participants. Further analysis was conducted to the point of theoretical saturation, whereby there were no advances on the established themes generated in the second tier of analysis [28]. Discussions were held via Skype to agree upon coding development and final analysis. Results of the thematic analysis of qualitative data are presented below.

## 3. Results

In total 30 interviews were conducted with Fijian students (15 male and 15 female) aged 14–17 years between September and October 2013. Overall, interview data revealed a predominantly coherent and interconnected series of key themes that were reiterated across the interviews: in particular, media preferences (visual, local), positive and negative tobacco promotions (point of sale, celebrity endorsements), and local tobacco trade (economic benefits of tobacco).

### 3.1. Smoking Knowledge and Attitudes

In general, most participants were well aware of the pervasive use of tobacco in Fiji society and able to describe some of the effects of tobacco: the link with “sickness” and death being the most widely mentioned. Second-hand smoke effects were also mentioned.

*“…it's not a good thing; it affects our health so why are they still selling it and the public why are they buying it because it can cause sickness and death because we never know when it's going to end our life.”*

* “if a lady is pregnant and might you know like somebody is smoke, it might smell and unborn baby will be…and that is bad.”*

Further, the impact on sporting ability, a highly cherished attribute among young Fijians, was also noted as a detrimental effect of using tobacco.

*“For sports we can really tell if a person in sports gets really tired easily you can tell because when they want to run around the ground they get tired easily it shows that they smoke too much and it effects there health.”*

Despite concerns about the health effects of tobacco use, smoking was widely accepted as a means of reducing stress, most often stress from work. Although several participants spoke about observing family members smoking as a means of relieving stress, the paradox of smoking leading to health stressors was also mentioned by some participants.

*“Some people say it takes the stress away. The disadvantage like it's not good especially to our health. In my family, you know from my father side, they smoke eh, sometimes I don't like it but you know they are adults. When I see children close to them, they still smoking…in my heart, it doesn't go well with me. Yeah…advantage people see that it take[s] the stress away, tobacco is not good.”*

*“Umm well somehow it's good and somehow it's bad in good it takes away the stress and in a bad way it can cause sickness and it can affect our health and we can die from taking it.” *

*“It helps you to put away your stress.” *

* “The negative is that it's very bad on young people that are schooling in secondary school and when they at home the go to the shop to buy cigarettes to probably get off sum stress or to get off from their parents and the positive working people I think it's relaxation for them.”*

Others spoke of the problem of “overusing” tobacco, suggesting a perception of a safe level or threshold of danger in tobacco use. In addition, it was evident that tobacco use was considered to have a positive social or mental health effect among adults, primarily as a leisure and stress-relief activity.

*“But for the disadvantage of it is that most people misuse it like used too much of it and they get sick.”*

*“Students will be using it for leisure activities and the bad thing is that it's good for adults.” *

The influence of others, both parents and young people, smoking was noted by several participants. In addition, the link between tobacco use and “becoming famous” was more likely to have been initiated, or reinforced at least, via media representations, where tobacco is almost exclusively presented in a positive light. Family use of tobacco was associated with relaxation and socialising.

*“Sometimes it starts from the parents, parents who smoke in front of their kids think that it's ok to smoke but sometimes it's about peer pressure when you get into wrong groups they want to do wrong things so they can be famous and they force you to do it and sometimes you think your being forced you think that you will become famous.” *

*“But for the disadvantage of it is that most people misuse it like used too much of it and they get sick. And when you sitting around like for me I usually sit with my parents and my…some of our friends they come in, they come drink grog with my parents like they sit around, most of them they are older than me, but they smoke and they do….”*

### 3.2. Media Preferences

The majority of young people interviewed valued television over almost all other media platforms. The relative simplicity and accessibility of televised information and the focus on “sound bites” in television combined with the immediate connectivity to visual media made it the medium of choice. Television was highly regarded for the entertainment qualities and the ease of access. Information presented on screen is perceived as easily digestible, visually stimulating segments which require less effort to access and interpret. However, it was also evident that the Internet was similarly valued but for the capacity to provide a greater depth of information to extend interests.

* “to be honest, television would be my preferred type of entertainment because it consists of many types of entertainment, such as like cartoons or news channel and sports compared to radio, there's not much information on the radio.” *

*“To me my favorite is not internet its television because television is a visible thingy where you get to see it, and hear it and find it colorful and attractive.”*

Although Internet connectivity in Fiji has improved vastly, it can still be affected by technological issues which disrupt access. Despite this issue, participants preferred the Internet and in particular social media for connecting with friends. Facebook, predictably, was the most widely accessed and preferred social networking site among the participants. The Internet was also cited as the preferred source of information (rather than pure entertainment) due to the depth and the selective capacity of the resource.

*“Umm I prefer to get it from websites and from the internet about events and people I get to know about them and what they doing.”*

*“Ok to be honest it would be Facebook I know it is not a good thing to be always online because Facebook has advantages and disadvantages well I think it's better for us to have access to the internet because it gives us more information and the background on what's actually happening.”*

### 3.3. Pro- and Antitobacco Media Portrayals

Tobacco-related imagery was more likely to be seen in connection with point of sale advertising and branding. Graphic warnings were thought to be effective strategy to shock young people into abstinence. The reaction to graphic warnings was predominantly expressed in terms of arousing disgust and fear that smoking tobacco kills and negatively effects health.

*“I thought of cancer… I think it scares children not to use it…I think it's a good way…I prefer looking at that, I prefer them selling it with the pictures.”*

*“Uhm…I don't usually, they don't usually advertise it like very often but it comes in like once in a blue moon. Like for me the advertisement is only on the packets of cigarettes.”*

The impact of point of sale and retail advertising was acutely apparent to some participants and this space was considered highly influential. The majority of participants considered the current antitobacco advertisements at shops to be effective, with some suggesting that more were needed to push the message further. A few participants felt that any images of smoking, even negative portrayals, were potentially conducive to promoting smoking, by simply drawing attention to the activity or as a personal reactionary (contrary) response to the image.

* “I think that they actually sending a message that's encouraging people to come buy the cigarettes. If I go to a shop and see those advertisements on the shops I would not think it's a good thing because it's telling people to come buy it when it's actually bad for them.” Another interviewee reported that despite having advertising on counters, people still buy and its individuals choice “it's your own choice if you buy it or not.” *

*“Somehow it won't really help us because the advertisements that are being advertised are not that much our age the students we don't really care about the advertisement we just care about other friends being forced.” *

Although there was some speculation that mass media channels such as the television were not likely vehicles for promoting tobacco, the Internet was framed as an important source. Several participants felt that advertising on the Internet has the potential to provide more opportunities to persuade young people to smoke, possibly via repeat screenings and interactive applications and platforms. Internet advertising was generally perceived in a negative light in terms of the potential impact on young people. Links to entertainment news and blogs about celebrity lifestyles, which included smoking imagery, were thought to “send the wrong message” to young people. As respecting adults and celebrities are critical to young Fijians, students found it contradictory when well-known sports stars promoted tobacco products.

*“Uhm…sometimes as you said how it can be linked to famous people…uhm…most of my cousins are die hard rugby fans so if they find out that like one of their rugby hero maybe linked to smoking, they will probably started just to be like that person, cause that's something like we young people like, try to be like other people…it has a huge impact on us.” *

Some students noted the tension that presents when sporting celebrities smoke—the erroneous message that smoking is acceptable versus the health impacts of tobacco on sporting abilities. Although Fiji has banned tobacco sponsorship of sporting events, study participants continued to make the connection between tobacco and sport.

### 3.4. Economic Benefits of Tobacco

Paradoxically, tobacco companies were considered an asset to Fiji's economy as many families were thought to be directly benefiting from sales or were indirectly benefiting from the “stress relief” of smoking. The benefits were variously articulated in terms of benefits for the country's economy (which clearly had indirect impact on family) and for parents. For the majority, it was a double-edged sword: benefits and costs in terms of health effects. However, for many it was the short-term benefits of a better life via a richer economy that was preferential.

*“[the] good side is it brings income to Fiji.”*

*“I think the shop keeper only wants to earn but they don't care about other people's life. They selling cigarettes and factory, well we can't say anything about that why they produce their cigarette.”*

*“It has allowed people in Fiji to get more money by the people buying tobacco so more money coming in to the government…and the Yeah and bad side is it causes diseases and lung cancer and all.”*

*“From the positive side of it, it brings a lot of income to Fiji so like our parents they get more pay….”*

## 4. Discussion

The purpose of the study was to examine how young people in Fiji view tobacco use in the context of social and family life and the media environment in Fiji. We identified several significant themes that appear to impact on how tobacco use was conceptualized. Despite a pervasive understanding that tobacco is harmful and that young people should not start, there were several areas of ambiguity and conflict in the understanding of tobacco use. Firstly, smoking was viewed as an acceptable adult choice and one that reduces stress levels among older members of the family. Tobacco control efforts have been noted in terms of point of sale education campaigns and graphic warning labels. Positive portrayals of tobacco in mainstream television media were not widely noted; however, the potential for the Internet to disseminate prosmoking images was evident. Previous research with Pacific populations suggests that although media is widely accessed and is likely to contribute to formation of expectations and desires about tobacco use, it is also likely to be an influential effect if it is consistent with familiar images of family and social life [28]. Finally, the sale of tobacco is perceived to have direct economic benefits for Fijian communities despite the well-established detrimental health effects.

The misperception that tobacco could be used in a rationed way and it was the* abuse* of tobacco that was the cause of most associated health problems. Strategies to realign this misperception (that a little tobacco is acceptable) are well documented [29, 30], but in the context of Fiji, also demand specific, locally relevant intervention. Indeed, despite the fact that the recently introduced graphic health warnings were generally well received, there appears to be a large gap in the knowledge and acceptance of tobacco control social marketing campaigns. Student comments that tobacco control messages, particularly those posted at point of sale, seem to reinforce that tobacco use is concerning. Warning about the dangers of tobacco use through persuasive public health messages is a cornerstone of comprehensive tobacco control [10]. Although potentially costly, televised social marketing campaigns have been proven to be an effective way to reduce tobacco use in high and low and middle income countries [31]. Adapting campaigns that have already been proven to work in other jurisdictions can reduce costs. Given student preferences for televised content, this presents an ideal opportunity to carry out similar graphic health warning campaigns as those proven to be effective in other Pacific settings, for example, Tonga [32]. Equally, although Internet access is less reliable in Fiji, and students reported a preference for television; web-based tobacco control social marketing campaigns hold promise being not only effective but less costly than television campaigns [33]. In a country and region where NCDs are at epidemic proportions, smart, relatively inexpensive, and impactful interventions tackling both policy and practice are essential.

Fiji as a middle income country remains vulnerable to domestic and regional economic, political, and climatic challenges [34]. Despite a strong tourism industry, fishing and mining development and steady income from overseas remittances, the economic outlook for the country is vulnerable [34]. In a small country, the impact of economic and political instability is profound, a fact observed in the shared narrative across the sample. British American Tobacco (BAT), the leading tobacco supplier for Fiji, has a vested interest in building market share in developing countries to compensate for declining revenue from developed countries where the appetite for tobacco is waning [14]. As part of their marketing strategy, BAT is actively reshaping views on the economic benefits of tobacco farming [35, 36]. As noted in a 2010 report, “tobacco farming is increasingly considered a profitable alternative to other agricultural crops as it provides a guaranteed, stable market and a steady source of income” [35]. It was evident that young Fijians we interviewed were acutely aware of the broader economic challenges within their country and the impact of supporting local economy has on livelihood. Some cynicism was expressed as to who actually benefits; however, this view was less commonly expressed than the former.

Bias in qualitative research is an inherent challenge due to the smaller sample size, in-depth but reflexive approach to interviewing and community (network) participants. Qualitative research methods are an appropriate and sympathetic research method to systematically elicit and interpret small group or individual perspectives. Our research is subject to several areas of potential bias. First, it is important to be reminded that our results reflect the views and perceptions of a small sample of young people who attend a central Suva school; they are therefore not generalizable to all Fijian young people. In addition, we sampled one school, albeit a central Suva school with a large multiethnic population. Few restrictions were placed on the selection of students, except that the school should be central and include a mix of students in terms of ethnicity, gender, and financial background. The same was applied when choosing the class that participated in the study. Finally, the interviews were conducted in English and were collected solely via one-to-one interviews. Although the majority of Fijian secondary school students are bilingual, we conducted the interviews in English which may have produced bias in the reporting from participants and interpretation by the researchers.

## 5. Conclusion

With high rates of tobacco use among adults in Fiji and evidence of an ambivalent view about tobacco use (for benefits of stress relief and economic benefits for the country) there are both real challenges and opportunities ahead for progressing tobacco control in Fiji. Continued effort at a policy level is imperative to providing an environment that promotes smoking denormalization. Part of this effort includes increasing taxation and smokefree environments and persisting with antitobacco marketing to counter misconceptions about benefits of tobacco use and reinforcing existing knowledge among young people of the harms of tobacco. With these measures in place, the likelihood broader social and individual behavior change can take place. In essence, we noted incongruity about the place tobacco holds in Fiji society, and without change at the broader social knowledge level, behaviors change is a challenge. However, there are positive signs of change, with young people welcoming antitobacco messages; this is an important step to population level behavior change.

Finally, there was a misperception that tobacco could be used in a responsible or reasonable way and it was the abuse of tobacco that was the cause of most associated health problems. Despite the fact that the recently introduced graphic health warnings were generally well received, there appears to be a large gap in the knowledge and acceptance of tobacco control social marketing campaigns. Student comments that tobacco control messages, particularly those posted at point of sale, seem to reinforce that tobacco use is concerning. Similarly, the understanding that “overusing” tobacco is a problem (suggesting a belief in a relatively safe level of use) is also problematic. This provides the opportunity to reinforce warning about the dangers of* any* tobacco use through persuasive public health messages alongside increasing the coherency of strong clear message consistent with the regions objectives of a Tobacco Free Pacific.
